# Helping the helpers: polyamines help maintain helper T-cell lineage fidelity

**DOI:** 10.1097/IN9.0000000000000002

**Published:** 2022-08-05

**Authors:** Tracy Murray Stewart, Cassandra E. Holbert, Robert A. Casero

**Affiliations:** 1 Department of Oncology, Johns Hopkins University School of Medicine and the Sidney Kimmel Comprehensive Cancer Center at Johns Hopkins, Baltimore, MD, USA

**Keywords:** polyamines, spermidine, spermine, hypusine, T cells, immune, histone acetylation

## Abstract

The awareness that polyamines play a critical role in immune system regulation and function is coming into focus as the biological systems and analytical tools necessary to evaluate their roles have become available. Puleston et al have recently demonstrated that polyamine metabolism plays a central role in helper T-cell lineage determination through the production of the translational cofactor hypusinated eIF5A and faithful epigenetic regulation through proper histone acetylation. Their findings add to the rapidly growing body of data implicating properly controlled polyamine metabolism as essential for a normally functioning immune system.

The naturally occurring mammalian polyamines, spermidine, and spermine, and their diamine precursor, putrescine, are required for growth, differentiation, and, in some cases, survival of normal cells and tissues. Dysregulation of polyamine metabolism is frequently observed in various pathologies ^[1,2]^. Polyamine functions include the maintenance of chromatin structure, facilitation of transcription and translation, regulation of ion channels, and scavenging of reactive oxygen species ^[3]^. Additionally, polyamines have long been postulated to play a role in the regulation of the immune system ^[4–6]^; however, only recently have the necessary tools become available to define their function more precisely. Although it is becoming clear, as will be seen below, that polyamines are necessary for normal immune system function, polyamines have also been implicated in the development of tumor-permissive microenvironments that facilitate the avoidance of immune detection and eradication of developing cancers ^[7]^. With several possible immune-modulating functions attributed to polyamines, it is important to more precisely define the molecular mechanisms by which polyamines affect immune cell function in both normal and disease states.

Puleston and colleagues, in *Polyamine metabolism is a central determinant of helper T-cell lineage fidelity* ^[8]^, have provided intriguing data as to how the polyamine spermidine regulates CD4^+^ T-cell proliferation and differentiation into appropriate lineages. First, the research team examined CD4^+^ T cells from wild-type (WT) mice and demonstrated that ornithine decarboxylase (ODC, Figure 1) and polyamine production increased during T-cell activation. ODC catalyzes the first rate-limiting step in mammalian polyamine biosynthesis to produce putrescine, and its activity is essential for the normal proliferation and differentiation of many normal cells and tissues ^[9]^. Interestingly, when the cells were polarized into different subsets of T helper (T_H_) cells (T_H_1, T_H_2, T_H_17, and T_regs_) in vitro, not only was ODC expression increased, but both spermidine and spermine synthases (the sequential enzymes that produce the higher polyamines from putrescine) also increased. These results suggest an increased requirement for polyamines for proper differentiation of T_H_ cells.

**Figure 1.**
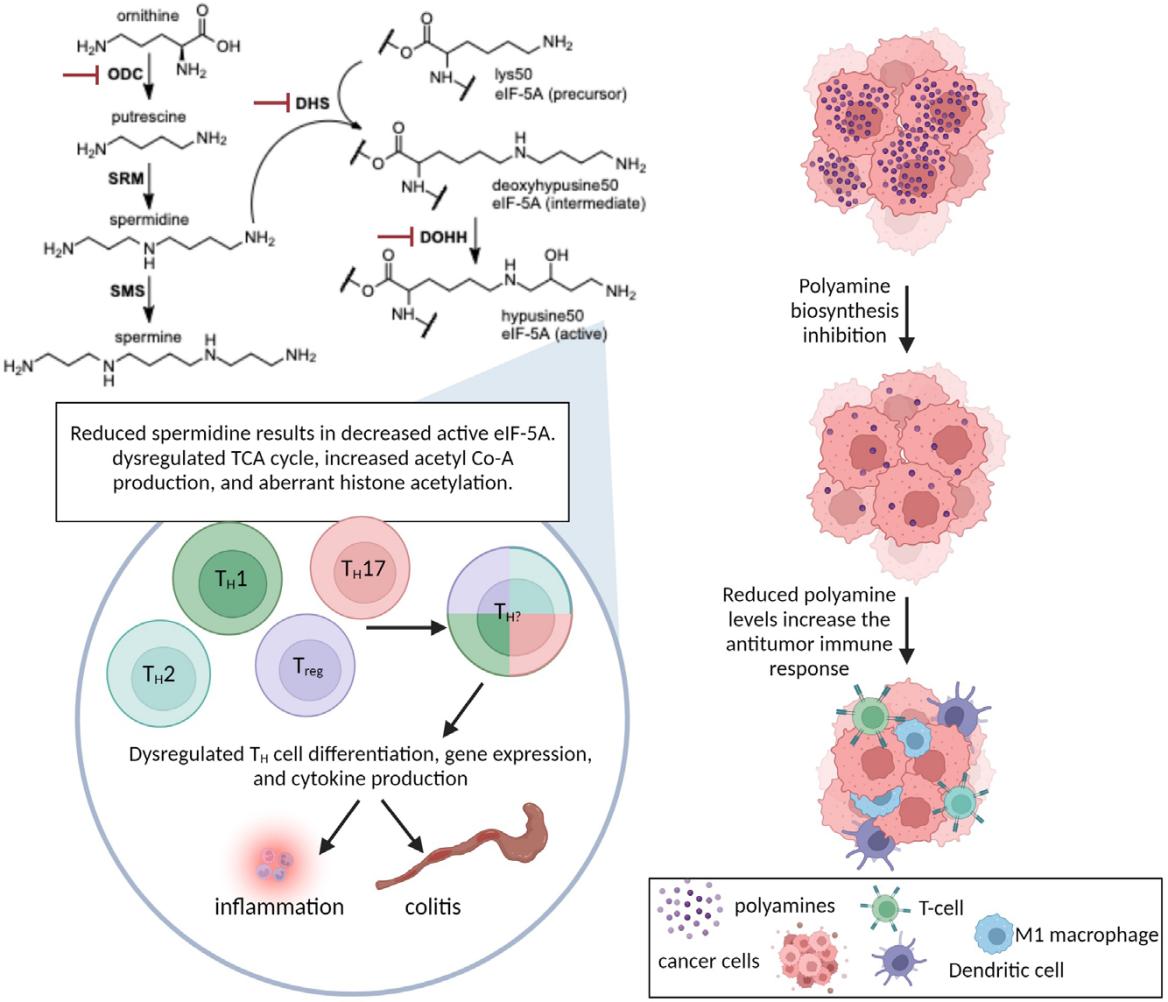
Effects of loss of polyamines on T-cell lineage fidelity and antitumor response. Polyamine biosynthesis starts with the decarboxylation of ornithine by ODC. Spermidine is synthesized by the addition of an aminopropyl group to the N^1^ of putrescine by SRM, and spermine is synthesized by the addition of an aminopropyl group to the N^8^ of spermidine by SMS. Fully active eIF-5A must first be hypusinated on its lysine 50 residue. The aminobutyl donor for the formation of hypusine is spermidine. The aminobutyl moiety is first transferred to lysine 50 of eIF-5A by DHS, and the mature protein is made by DOHH. DHS, deoxyhypusine synthase; DOHH, deoxyhypusine hydroxylase; eIF-5A, eukaryotic initiation factor 5A; ODC, ornithine decarboxylase; SMS, spermine synthase; SRM, spermidine synthase.

To determine if increased polyamine production was required for T_H_ cell differentiation, the authors compared WT mice with those containing specially engineered ODC-deficient T cells (*Odc-∆T*). T cells from both WT and *Odc-∆T* mice produced comparable numbers of CD8^+^ and CD4^+^ ratios in the spleen. However, after activation, the *Odc-∆T* cells had significantly reduced polyamine levels compared with WT cells, and the CD4^+^ T cells from the *Odc-∆T* mice exhibited altered differentiation and cytokine production. Specifically, when provided with the same stimuli to differentiate as the WT CD4^+^ T cells, the *Odc-∆T* cells exhibited a dysregulated expression of cytokines. Although the T_H_1-differentiated cells produced the canonical T_H_1 cytokine IFNγ, they also aberrantly produced the T_H_17 cytokine IL-17A. Potentially more significantly, all *Odc-∆T* T_H_ subsets produced the proinflammatory cytokines IFNγ and IL-13. Interestingly, the expression of IL-17A in T_H_17 cells was significantly reduced.

To better understand the reason for this aberrant cytokine expression in the T_H_ lineages of *Odc-∆T* cells, the investigators determined the expression of T-cell lineage-specific transcription factors following exposure of the *Odc-∆T* to polarizing conditions. The results of these studies indicated that the aberrant expression of cytokines resulted from the unusual expression of normally lineage-specific transcription factors under polarizing conditions in which they would typically not be expressed. For example, T-bet, a T_H_1 transcription factor, was also expressed in T_H_2, T_H_17, and T_regs_, and the T_H_2-specific transcription factor GATA3 was increased in T_H_1 and T_H_17 cells. This alteration of transcription factor expression underscores the important role polyamines play in T-cell lineage determination.

To confirm the necessity of polyamines in lineage determination in vivo, naïve CD4^+^ T cells from WT and *Odc-∆T* mice were injected into T-cell-deficient Rag1^−/−^ mice. After 36 days, the mice receiving the *Odc-∆T* cells had to be sacrificed due to weight loss and diarrhea resulting from colonic inflammation. In those mice, there was an increase in T-cell IFNγ expression but a decrease in cells expressing IL-17A. These studies thus confirmed the necessity of polyamines for proper fidelity of T_H_ cells.

Although, as stated above, polyamines are required for many normal cellular processes, there is only one known protein, the translational elongation factor eIF5A, that requires the polyamine, spermidine, for an essential, post-translational modification ^[10]^. Originally thought to be a eukaryotic translational initiation factor, subsequent studies confirmed that eIF5A is a translational cofactor necessary to prevent ribosomal stalling on difficult-to-translate mRNAs. For proper function, eIF5A must first be hypusinated through a two-step process that uses spermidine to modify a specific lysine (K-50) on eIF5A (Figure 1) ^[11]^. The first step in this lysine modification is the transfer of the aminobutyl moiety from spermidine by deoxyhypusine synthase (DHS), producing eIF5A-deoxyhypusine. The second step, catalyzed by deoxyhypusine hydroxylase (DOHH), generates the mature, hypusinated eIF5A. To determine if this was the critical mechanism linking polyamines to CD4^+^ T-cell lineage fidelity, the authors generated *Dhs-∆T* and *Dohh-∆T* mice to produce CD4^+^ T cells without the ability to generate the hypusinated form of eIF5A. In each case, the CD4^+^ T cells from engineered mice behaved similarly to the *Odc-∆T* cells when induced to differentiate in vitro, expressing high levels of IFNγ and IL-17A. Additionally, the loss of either enzyme in vivo resulted in significant T-cell dysregulation leading to inflammation and colitis.

It had been determined that T-cell lineage determination is regulated by epigenetic mechanisms, including chromatin modifications ^[12]^, and polyamines have been shown to regulate histone acetylation ^[13]^. The authors next sought to determine if epigenetic regulation of T-cell gene expression was responsible for the dysregulated cytokine expression exhibited by the *Odc–*, *Dhs–*, and *Dohh–* T cells. Through a series of experiments to determine chromatin accessibility and histone protein modifications, it was determined that multiple histone marks were dysregulated in the *Odc–*, *Dhs–*, and *Dohh–* T_H_ cell subsets after differentiation, when compared with WT T_H_ cells. As these data indicated that chromatin architecture was indeed altered in response to perturbations in polyamine metabolism, and it is known that histone acetylation is associated with transcriptional activation, further experiments were performed that either pharmacologically inhibited histone acetylation or deleted proteins necessary for the acetylation of the transcriptional activating marks H3K27 and H3K9. In both cases, the reduction of acetylase activity restored a more normal transcriptional program to *Odc–* and *Dohh–* CD4^+^ T cells when stimulated.

Finally, using ^13^C-glucose tracing and confirming with genetic alterations, it was found that the tricarboxylic acid (TCA) cycle was perturbed in *Odc–* and *Dohh–* CD4^+^ T cells, resulting in increased levels of acetyl-CoA, the necessary source of acetyl groups used by histone acetyltransferases in the acetylation of histone protein. Thus, it is postulated that the increased acetyl-CoA contributes to the aberrant histone acetylation and cytokine expression in T_H_ cells with perturbed polyamine/hypusine metabolism.

Taken together, the data presented by Puleston et al ^[8]^ confirm the critical role that polyamine metabolism plays in proper lineage fidelity in T cells. Importantly, these data suggest mechanisms by which dysregulation of polyamine metabolism and thus T-cell regulation could lead to potential pathologies. An age-related decrease in polyamines, particularly spermidine, has been established by several studies ^[14–16]^. Multiple sequelae have been linked to such a decrease, mostly attributed to a decrease in autophagy ^[13]^. Decreased spermidine levels have been linked with autophagy-mediated immune senescence, manifesting as inefficient adaptive immune responses to stimuli such as vaccinations and infections in older individuals. Spermidine controlled autophagic flux in these systems via hypusinated eIF5A-mediated translation of TFEB, a master regulator of autophagosomal and lysosomal biogenesis ^[17,18]^ (note: studies indicating polyamine-induced autophagy should be carefully reviewed as some study designs have inherent problems that likely confound the interpretation of the results ^[19]^). Importantly, several studies have demonstrated that polyamines are effective in reducing inflammation ^[20–22]^. Consequently, an inflammatory phenotype resulting from dysregulation of T_H_ cell differentiation due to spermidine reduction may indicate a direct mechanism by which age-related increases in inflammation occur.

It is also important to note that T cells are not the only immune cells regulated by polyamines, as regulation of macrophage polarization has also been described ^[23]^. Hardbower et al demonstrated that engineered loss of ODC in macrophages leads to M1 polarization and increased inflammation of the stomach and colon ^[24]^. This M1 polarization was also accompanied by an increase in transcriptionally activating histone modifications including H3K9 acetylation and H3K4 monomethylation. Thus, similar to the results of Puleston et al ^[8]^, polyamine depletion in macrophages can lead to a pathogenic inflammatory response.

Travers et al also demonstrated a bias toward M1 polarization when ovarian tumor-bearing animals were treated with a combination of the ODC inhibitor, 2-difluoromethylornithine (DFMO), and the DNA methyltransferase inhibitor, 5-azacitidine. However, in this case, rather than inducing a pathogenic phenotype, this treatment regimen resulted in a decrease in tumor burden, and a greater antitumor immune response. Similarly, other studies have demonstrated that polyamine blocking therapy, the process of inhibiting both polyamine biosynthesis and polyamine import, can produce significant antitumor immune responses, thus opening several potential avenues for the successful treatment of neoplastic disease ^[25,26]^. These studies, among others, illustrate the importance of polyamines in maintaining a tumor-permissive immune microenvironment ^[7]^.

Overall, the above studies and many others underscore the critical roles that polyamines and their metabolism play in the regulation of the immune system ^[27]^. Importantly, depending on the specific setting, changes in polyamine metabolism can lead to deleterious effects, or its modulation can be used for therapeutic advantage. Further studies will be required to gain a better understanding of the intricacies of polyamine influences on the immune system. Such studies will provide the necessary information to not only address age-related pathologies associated with decreased polyamines and increased inflammation but also to allow the development of potentially more efficacious cancer treatments.

## Conflicts of interest

Work in the Casero Laboratory is funded, in part, by a sponsored research agreement with Panbela Therapeutics, a company interested in the development of therapeutics targeting polyamine metabolism.

## Funding

Work in the Casero laboratory is supported by grants from the US National Institutes of Health (R01CA204345 and R01CA235863), the Samuel Waxman Cancer Research Foundation, the University of Pennsylvania Orphan Disease Center Million Dollar Bike Ride (MDBR-20-135-SRS), the Chan Zuckerberg Initiative and a research contract with Panbela Therapeutics Inc.
